# 

**Published:** 1921-02-05

**Authors:** 


### Forthcoming Events.

Medical Officers of Schools Association.—General Meeting, 11 Chandos Street, Cavendish Square, W. 1, Tuesday, February 15, 1921, 4.45 p.m. Dr. Elwin H. T. Nash will read a paper on "School Punishments."

### Gifts and Bequests.

Mr. H. L. Heach, of Worcester, has left £300 to Worcester Infirmary.

Mr. E. F. Ward, of Stockport, left £500 railway stock to the Stockport Infirmary.

Miss Mary Hannah, of Carlisle, has left £250 to the Cancer Research Committee.

Miss Anne Haworth, of Accrington, has left £5,000 to Accrington Victoria Hospital.

The British Red Cross Society has made a further grant of £25,000 to the Village Centres Council.

The Royal Hospital for Incurables, Putney, has been left £100 under the will of the late Mr. J. R. Burrows, of New Cross.

The Empress Eugenie has left 20,000 francs each to the French Hospital in London and to Queen Mary's Auxiliary Hospital at Roehampton.

As a result of the Armistice Sunday appeal for the Red Cross, £448 has been received from Beira, South Africa, and £800 from Canton, China.

Salford Royal Hospital receives £105 under the will of Alderman Samuel Rudman, and Southport Infirmary £50, left by Mrs. Harrott Harper.

The Royal Hospital for Children and Women, Waterloo Road, has received £500 from the discretionary bequest made by the late Sir Frederick Cook.

Mr. John Molloy, of Sheffield, left £100 each to the Sheffield Royal Infirmary, the Royal Hospital, the Jessop Hospital for Women, and the Children's Hospital.

The late Sir Riley Lord, of Newcastle-on-Tyne, has left his mansion to the Royal Infirmary, to be used as a convalescent home, together with £5,000 for upkeep and £1,000 for a cot.

The Royal National Hospital for Consumption (Ireland), Newcastle, County Wicklow, has been left £300 by Mr. A. E. West, a well-known judge and breeder of hunters and bloodstock.

The workpeople of Messrs. W. D. and H. O. Wills have distributed during 1920 £2,044 amongst Bristol charities, including £800 to the General Hospital and £400 to the Royal Infirmary.

Mr. A. H. Wilson, a director of Messrs. Straker Bros., Ltd., stationers, has left £250 each to the General Hospital, the Eye and Ear Hospital, and the Homœopathic Hospital, all at Tunbridge Wells.

The ultimate residue of the estate of Mr. Herbert Ward, of Sutton Coldfield, is left to seven institutions, including the General Hospital, Queen's Hospital, and Children's Hospital, Birmingham.

£500 each has been left to the Cranleigh Village Hospital and the Royal Surrey County Hospital, Guildford, and £1,000 to the Montreal General Hospital under the will of Mr. Sherringham Shepherd.

The residue of the property of Mr. B. Duncan Gibb, of Battle (about £20,000), is to be divided between St. Thomas's, Guy's, St. Bartholomew's, the Middlesex, and the London Hospitals and Earlswood Asylum.

Under the will of Mr. Christopher Collins, of Solihull, Warwick, the ultimate residue is destined to be divided between the Leamington Home for Incurables, the Birmingham Children's Hospital, and the Birmingham University.

Mrs. B. M. Haynes, late of Sheffield, has bequeathed £1,000 to the Sheffield Royal Hospital for the endowment of a cot in memory of her brother, Noel Wilcock, killed in the recent war. The residue of the estate is to be divided between the Royal Hospital, Royal Infirmary, and the Jessop Hospital for Women, Sheffield.

The late Mr. Thomas Leonard, of Dunsany, Co. Meath, who left £378,732, has bequeathed £10,000 to the Mater Misericordiæ Hospital, Dublin, and £5,000 to the Royal Hospital for Incurables. Half the residue of his estate, which is expected to amount to £100,000, is bequeathed to such charities as he should direct or his executors should choose.

